# Ethyl 3,4-dimethyl-1*H*-pyrrole-2-carboxyl­ate

**DOI:** 10.1107/S160053681003179X

**Published:** 2010-08-18

**Authors:** Wei-Na Wu, Xiao-Xia Li, Qiu-Fen Wang, Yan-Wei Li

**Affiliations:** a Department of Physics and Chemistry, Henan Polytechnic University, Jiaozuo 454000, People’s Republic of China; b Institute of Functional Materials, Jiangxi University of Finance & Economics, Nanchang 330013, People’s Republic of China

## Abstract

The non-H atoms of the title compound, C_9_H_13_NO_2_, are almost coplanar (r.m.s. deviation = 0.0358 Å). Weak inter­molecular N—H⋯O hydrogen bonds link the mol­ecules into zigzag chains along the *b* axis with graph-set motif *C*(5). The chains are further linked into a three-dimensional network by C—H⋯O hydrogen bonds and C—H⋯π inter­actions.

## Related literature

Schiff bases containing pyrrole units have been extensively investigated due to their excellent coordination abilities, see: Wu *et al.* (2003 ▶). For our studies on bis­(pyrrol-2-yl-methyl­ene­amine) ligands, see: Wang *et al.*, (2008 ▶). For a similar structure, 5-formyl-3,4-dimethyl-1*H*-pyrrole-2-carboxyl­ate, see Wu *et al.* (2009 ▶). For the preparation, see: Helms *et al.* (1992 ▶). For graph-set motifs, see: Etter *et al.* (1990 ▶).
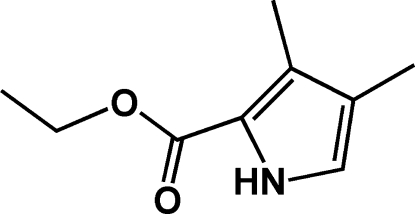


## Experimental

### 

#### Crystal data

C_9_H_13_NO_2_
*M* *_r_* = 167.20
Monoclinic, 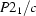

*a* = 7.7485 (2) Å
*b* = 7.0611 (2) Å
*c* = 17.2167 (5) Å
β = 95.103 (2)°
*V* = 938.24 (5) Å^3^
*Z* = 4
Mo *K*α radiation
μ = 0.08 mm^−1^
*T* = 296 K
0.28 × 0.26 × 0.18 mm

#### Data collection

Bruker SMART APEX CCD diffractometer
Absorption correction: multi-scan (*SADABS*; Bruker, 2007 ▶) *T*_min_ = 0.977, *T*_max_ = 0.985
8174 measured reflections
2146 independent reflections
1579 reflections with *I* > 2σ(*I*)
*R*_int_ = 0.019

#### Refinement

*R*[*F* ^2^ > 2σ(*F* ^2^)] = 0.044
*wR*(*F* ^2^) = 0.136
*S* = 1.04
2146 reflections
112 parameters
H-atom parameters constrained
Δρ_max_ = 0.21 e Å^−3^
Δρ_min_ = −0.17 e Å^−3^

### 

Data collection: *APEX2* (Bruker, 2007 ▶); cell refinement: *SAINT* (Bruker, 2007 ▶); data reduction: *SAINT*; program(s) used to solve structure: *SHELXS97* (Sheldrick, 2008 ▶); program(s) used to refine structure: *SHELXL97* (Sheldrick, 2008 ▶); molecular graphics: *SHELXTL* (Sheldrick, 2008 ▶); software used to prepare material for publication: *SHELXTL*.

## Supplementary Material

Crystal structure: contains datablocks I, global. DOI: 10.1107/S160053681003179X/fb2205sup1.cif

Structure factors: contains datablocks I. DOI: 10.1107/S160053681003179X/fb2205Isup2.hkl

Additional supplementary materials:  crystallographic information; 3D view; checkCIF report

## Figures and Tables

**Table 1:** Hydrogen-bond geometry (Å, °) *Cg*1 is the centroid of the N1,C1–C4 ring.

*D*—H⋯*A*	*D*—H	H⋯*A*	*D*⋯*A*	*D*—H⋯*A*
N1—H1⋯O1^i^	0.86	2.13	2.9264 (16)	154
C4—H4⋯*Cg*1^ii^	0.93	2.92	3.7520 (17)	149
C9—H9*A*⋯*Cg*1^iii^	0.96	2.86	3.650 (2)	141

Symmetry codes: (i) 
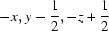
; (ii) 
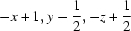
; (iii) 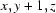.

**Fig. 1.**
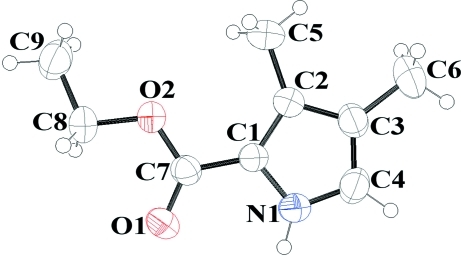
The title molecule with the displacement ellipsoids shown at the 50% probability level.

**Fig. 2.**
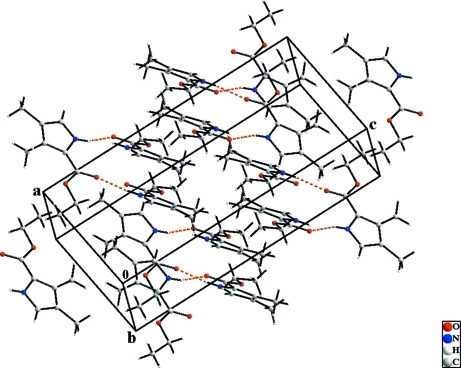
The crystal packing for the title compound via N—H···O hydrogen bonds shown as the dashed lines.

**Fig. 3.**
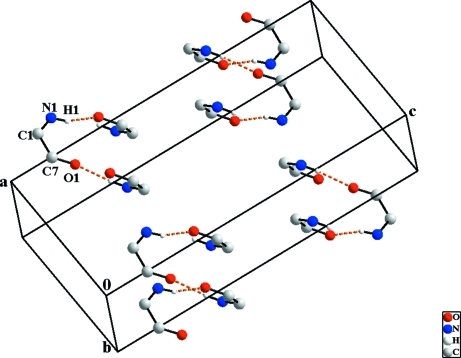
A view showing zig-zag chains with the graph-set motifs C(5) pertinent to the N—H···O hydrogen bonds (the dashed lines) in the title structure. The atoms not involved in this motif have been omitted for clarity.

## supplementary crystallographic information

### Comment

Schiff bases containing pyrrole units have been extensively investigated due to their excellent coordination abilities (Wu *et al.*, 2003). As a part of our studies on bis(pyrrol-2-yl-methyleneamine) ligands (Wang *et al.*, 2008), the crystal structure of the title compound is reported here.

The non-hydrogen atoms of the title molecule (Fig. 1) are situated in a fair plane (r.m.s. deviation of the non-hydrogen atoms being 0.0358 Å). In the crystal structure, the molecules are linked by weak intermolecular N—H···O hydrogen bonds, forming zig-zag chains with the graph-set motifs C(5) (Etter & MacDonald, 1990). The chains are extended along the *b* axis (Tab. 1, Fig. 2, Fig. 3). The structure is also stabilized by the C—H···O hydrogen bonds (Tab. 1) and C—H···π-electron ring interactions (Tab. 1).

### Experimental

The title compound was prepared according to Helms *et al.* (1992). Acetic acid (114 ml) was placed in a 1-*L* round-bottom flask and heated to 85 °C. Sodium acetate (31.09 g), 27.54 g of sodium 2-methyl-3-oxo-l-butene-1-oxide, 37.20 g of diethyl 2-(hydroxyimino)malonate, and a solution of 47 ml of acetic acid in 19.6 ml of H_2_O were then added in the respective order. The reaction temperature was raised to 95 °C, and 43.26 g of Zn-dust was added over 45 min while maintaining the temperature between 95 and 110 °C. After the addition of Zn-dust had been completed, the mixture was stirred while keeping its temperature at 110 °C for further 45 min. The reaction mixture was then poured into 500 ml of ice water. The obtained solid was filtered, washed with water and subsequently dissolved in dichloromethane. The solution was washed with saturated sodium hydrogencarbonate, dried with anhydrous sodium sulfate and then the solvent was removed under reduced pressure. The crude product was purified by column chromatography on a silica gel [*R*_f_ = 0.68, petroleum ether-ethyl acetate (100:1) as an eluent] to yield 4.82 g (13%) of the title compound. Colourless block crystals [average size: 0.25× 0.25 × 0.20 mm] were obtained by slow evaporation of the ethyl acetate solution at room temperature.

### Refinement

All the H atoms were located in the difference electron density map. The H atoms were situated into the idealized positions with the carrier atom-H distances = 0.93 Å for aryl, 0.97 for methylene, 0.96 Å for the methyl and 0.86 Å for the secondary amine hydrogens. The *U*_iso_ values were constrained to be 1.5*U*_eq_ of the carrier atom for the methyl H atoms and 1.2*U*_eq_ for the remaining H atoms.

### Crystal data

**Table:**

C_9_H_13_NO_2_	*F*(000) = 360
*M**_r_* = 167.20	*D*_x_ = 1.184 Mg m^−^^3^
Monoclinic, *P*2_1_/*c*	Mo *K*α radiation, λ = 0.71073 Å
Hall symbol: -P 2ybc	Cell parameters from 3131 reflections
*a* = 7.7485 (2) Å	θ = 2.4–24.8°
*b* = 7.0611 (2) Å	µ = 0.08 mm^−^^1^
*c* = 17.2167 (5) Å	*T* = 296 K
β = 95.103 (2)°	Block, colourless
*V* = 938.24 (5) Å^3^	0.28 × 0.26 × 0.18 mm
*Z* = 4	

### Data collection

**Table:**

Bruker SMART APEX CCD diffractometer	2146 independent reflections
Radiation source: fine-focus sealed tube	1579 reflections with *I* > 2σ(*I*)
graphite	*R*_int_ = 0.019
φ and ω scans	θ_max_ = 27.5°, θ_min_ = 2.4°
Absorption correction: multi-scan (*SADABS*; Bruker, 2007)	*h* = −9→10
*T*_min_ = 0.977, *T*_max_ = 0.985	*k* = −9→9
8174 measured reflections	*l* = −22→22

### Refinement

**Table:**

Refinement on *F*^2^	Primary atom site location: structure-invariant direct methods
Least-squares matrix: full	Secondary atom site location: difference Fourier map
*R*[*F*^2^ > 2σ(*F*^2^)] = 0.044	Hydrogen site location: difference Fourier map
*wR*(*F*^2^) = 0.136	H-atom parameters constrained
*S* = 1.04	*w* = 1/[σ^2^(*F*_o_^2^) + (0.0691*P*)^2^ + 0.1432*P*] where *P* = (*F*_o_^2^ + 2*F*_c_^2^)/3
2146 reflections	(Δ/σ)_max_ < 0.001
112 parameters	Δρ_max_ = 0.21 e Å^−^^3^
0 restraints	Δρ_min_ = −0.17 e Å^−^^3^
49 constraints	

### Special details

**Table:**

Geometry. All e.s.d.'s (except the e.s.d. in the dihedral angle between two l.s. planes) are estimated using the full covariance matrix. The cell e.s.d.'s are taken into account individually in the estimation of e.s.d.'s in distances, angles and torsion angles; correlations between e.s.d.'s in cell parameters are only used when they are defined by crystal symmetry. An approximate (isotropic) treatment of cell e.s.d.'s is used for estimating e.s.d.'s involving l.s. planes.
Refinement. Refinement of *F*^2^ against ALL reflections. The weighted *R*-factor *wR* and goodness of fit *S* are based on *F*^2^, conventional *R*-factors *R* are based on *F*, with *F* set to zero for negative *F*^2^. The threshold expression of *F*^2^ > σ(*F*^2^) is used only for calculating *R*-factors(gt) *etc*. and is not relevant to the choice of reflections for refinement. *R*-factors based on *F*^2^ are statistically about twice as large as those based on *F*, and *R*- factors based on ALL data will be even larger.

### Fractional atomic coordinates and isotropic or equivalent isotropic displacement parameters (Å^2^)

**Table:**

	*x*	*y*	*z*	*U*_iso_*/*U*_eq_	
O2	0.14704 (13)	0.48430 (14)	0.11453 (6)	0.0534 (3)	
O1	−0.00527 (14)	0.33077 (15)	0.19987 (7)	0.0615 (3)	
N1	0.24389 (16)	0.04205 (17)	0.20998 (7)	0.0498 (3)	
H1	0.1597	0.0168	0.2377	0.060*	
C2	0.41028 (18)	0.1886 (2)	0.12961 (8)	0.0463 (4)	
C7	0.12075 (18)	0.34064 (19)	0.16247 (8)	0.0445 (3)	
C1	0.25621 (17)	0.20054 (19)	0.16438 (8)	0.0422 (3)	
C8	0.0178 (2)	0.6323 (2)	0.10833 (10)	0.0582 (4)	
H8A	0.0125	0.6946	0.1583	0.070*	
H8B	−0.0953	0.5796	0.0923	0.070*	
C4	0.3843 (2)	−0.0672 (2)	0.20438 (10)	0.0561 (4)	
H4	0.4060	−0.1820	0.2299	0.067*	
C3	0.49035 (19)	0.0178 (2)	0.15508 (9)	0.0521 (4)	
C9	0.0697 (3)	0.7705 (3)	0.04886 (11)	0.0686 (5)	
H9A	0.1837	0.8179	0.0644	0.103*	
H9B	−0.0111	0.8738	0.0447	0.103*	
H9C	0.0699	0.7085	−0.0007	0.103*	
C5	0.4845 (2)	0.3287 (3)	0.07667 (11)	0.0702 (5)	
H5A	0.3973	0.3650	0.0363	0.105*	
H5B	0.5806	0.2725	0.0536	0.105*	
H5C	0.5234	0.4386	0.1060	0.105*	
C6	0.6620 (2)	−0.0563 (3)	0.13353 (13)	0.0784 (6)	
H6A	0.7528	0.0278	0.1533	0.118*	
H6B	0.6604	−0.0640	0.0778	0.118*	
H6C	0.6820	−0.1799	0.1557	0.118*	

### Atomic displacement parameters (Å^2^)

**Table:**

	*U*^11^	*U*^22^	*U*^33^	*U*^12^	*U*^13^	*U*^23^
O2	0.0535 (6)	0.0449 (6)	0.0639 (7)	0.0109 (4)	0.0178 (5)	0.0096 (5)
O1	0.0585 (7)	0.0564 (7)	0.0742 (8)	0.0072 (5)	0.0314 (6)	0.0035 (5)
N1	0.0523 (7)	0.0466 (7)	0.0523 (7)	0.0007 (5)	0.0151 (6)	0.0061 (5)
C2	0.0450 (7)	0.0479 (8)	0.0469 (8)	0.0013 (6)	0.0092 (6)	0.0019 (6)
C7	0.0464 (7)	0.0416 (7)	0.0467 (8)	−0.0002 (6)	0.0107 (6)	−0.0038 (6)
C1	0.0433 (7)	0.0403 (7)	0.0440 (7)	0.0001 (5)	0.0091 (6)	0.0021 (6)
C8	0.0595 (9)	0.0451 (8)	0.0711 (10)	0.0133 (7)	0.0119 (8)	0.0018 (8)
C4	0.0615 (9)	0.0468 (8)	0.0595 (9)	0.0088 (7)	0.0025 (7)	0.0079 (7)
C3	0.0462 (8)	0.0545 (9)	0.0560 (9)	0.0084 (6)	0.0059 (7)	−0.0004 (7)
C9	0.0832 (12)	0.0541 (10)	0.0685 (11)	0.0128 (9)	0.0066 (9)	0.0102 (9)
C5	0.0643 (10)	0.0732 (12)	0.0775 (12)	0.0024 (9)	0.0302 (9)	0.0191 (9)
C6	0.0571 (10)	0.0842 (13)	0.0950 (14)	0.0260 (9)	0.0134 (10)	0.0026 (11)

### Geometric parameters (Å, °)

**Table:**

O2—C7	1.3347 (17)	C4—C3	1.371 (2)
O2—C8	1.4446 (17)	C4—H4	0.9300
O1—C7	1.2186 (17)	C3—C6	1.506 (2)
N1—C4	1.3440 (19)	C9—H9A	0.9600
N1—C1	1.3752 (17)	C9—H9B	0.9600
N1—H1	0.8600	C9—H9C	0.9600
C2—C1	1.3849 (19)	C5—H5A	0.9600
C2—C3	1.409 (2)	C5—H5B	0.9600
C2—C5	1.494 (2)	C5—H5C	0.9600
C7—C1	1.4406 (19)	C6—H6A	0.9600
C8—C9	1.495 (2)	C6—H6B	0.9600
C8—H8A	0.9700	C6—H6C	0.9600
C8—H8B	0.9700		
			
C7—O2—C8	116.91 (12)	C4—C3—C2	107.21 (13)
C4—N1—C1	109.16 (12)	C4—C3—C6	126.27 (15)
C4—N1—H1	125.4	C2—C3—C6	126.51 (15)
C1—N1—H1	125.4	C8—C9—H9A	109.5
C1—C2—C3	106.86 (12)	C8—C9—H9B	109.5
C1—C2—C5	128.10 (13)	H9A—C9—H9B	109.5
C3—C2—C5	125.02 (13)	C8—C9—H9C	109.5
O1—C7—O2	122.94 (13)	H9A—C9—H9C	109.5
O1—C7—C1	124.41 (14)	H9B—C9—H9C	109.5
O2—C7—C1	112.65 (12)	C2—C5—H5A	109.5
N1—C1—C2	107.69 (12)	C2—C5—H5B	109.5
N1—C1—C7	118.98 (12)	H5A—C5—H5B	109.5
C2—C1—C7	133.32 (13)	C2—C5—H5C	109.5
O2—C8—C9	107.22 (13)	H5A—C5—H5C	109.5
O2—C8—H8A	110.3	H5B—C5—H5C	109.5
C9—C8—H8A	110.3	C3—C6—H6A	109.5
O2—C8—H8B	110.3	C3—C6—H6B	109.5
C9—C8—H8B	110.3	H6A—C6—H6B	109.5
H8A—C8—H8B	108.5	C3—C6—H6C	109.5
N1—C4—C3	109.09 (14)	H6A—C6—H6C	109.5
N1—C4—H4	125.5	H6B—C6—H6C	109.5
C3—C4—H4	125.5		
			
C8—O2—C7—O1	0.1 (2)	O1—C7—C1—C2	−177.02 (15)
C8—O2—C7—C1	179.83 (13)	O2—C7—C1—C2	3.3 (2)
C4—N1—C1—C2	−0.19 (16)	C7—O2—C8—C9	−177.04 (13)
C4—N1—C1—C7	−178.84 (13)	C1—N1—C4—C3	−0.06 (18)
C3—C2—C1—N1	0.37 (16)	N1—C4—C3—C2	0.29 (18)
C5—C2—C1—N1	−178.16 (16)	N1—C4—C3—C6	179.24 (16)
C3—C2—C1—C7	178.74 (16)	C1—C2—C3—C4	−0.40 (17)
C5—C2—C1—C7	0.2 (3)	C5—C2—C3—C4	178.18 (16)
O1—C7—C1—N1	1.2 (2)	C1—C2—C3—C6	−179.35 (16)
O2—C7—C1—N1	−178.46 (12)	C5—C2—C3—C6	−0.8 (3)

### Hydrogen-bond geometry (Å, °)

**Table:**

Cg1 is the centroid of the N1,C1–C4 ring.

**Table:**

*D*—H···*A*	*D*—H	H···*A*	*D*···*A*	*D*—H···*A*
N1—H1···O1^i^	0.86	2.13	2.9264 (16)	154.
C5—H5A···O2	0.96	2.60	2.962 (2)	103.
C4—H4···Cg1^ii^	0.93	2.92	3.7520 (17)	149
C9—H9A···Cg1^iii^	0.96	2.86	3.650 (2)	141

Symmetry codes: (i) −*x*, *y*−1/2, −*z*+1/2; (ii) −*x*+1, *y*−1/2, −*z*+1/2; (iii) *x*, *y*+1, *z*.
